# Endoscopic Optical Coherence Tomography (OCT): Advances in Gastrointestinal Imaging

**DOI:** 10.1155/2014/376367

**Published:** 2014-02-26

**Authors:** Tejas S. Kirtane, Mihir S. Wagh

**Affiliations:** ^1^Department of Medicine, Washington Hospital Center, 110 Irving St NW, Washington, DC 20010, USA; ^2^Division of Gastroenterology, University of Florida, 1600 SW Archer Road, P.O. Box 100214, Gainesville, FL 32610, USA

## Abstract

In the rapidly evolving field of endoscopic gastrointestinal imaging, Optical Coherence Tomography (OCT) has found many diverse applications. We present the current status of OCT and its practical applications in imaging normal and abnormal mucosa in the esophagus, stomach, small and large intestines, and biliary and pancreatic ducts. We highlight technical aspects and principles of imaging, assess published data, and suggest future directions for OCT-guided evaluation and therapy.

## 1. Introduction

Endoluminal imaging in gastrointestinal endoscopy has seen the advent of rapidly evolving new modalities over the last decade. Narrow band imaging [1], confocal laser endomicroscopy [2], and Optical Coherence Tomography (OCT or VLE: Volumetric Laser Endomicroscopy) [3] are some of the newer imaging techniques that have shown promise in the early detection of dysplasia and mucosal cancers and surveillance of cancers after endoscopic therapy. We present a practical assessment of OCT and its clinical applications focusing on recent advances in OCT in the diagnosis and management of gastrointestinal diseases.

## 2. OCT Technology

Conceptually, OCT (or Volumetric Laser Endomicroscopy/VLE) is analogous to B-mode ultrasonography, with the exception that near infrared light in the 700 to 1500 nm range of wavelength is used instead of sound waves to generate an image of the mucosal structure and its abnormalities using an interferometer device setup. Initially described in 1999 [4], OCT has evolved over the years to allow for higher resolution and rapid imaging. Time domain OCT was described initially but could not achieve high scanning rates. Subsequently, Fourier domain OCT was developed which allows for rapid image acquisition and generation of real time *in vivo* 2-dimensional and 3-dimensional mucosal renditions [5].

An OCT probe can be passed through the accessory channel of an endoscope and can be kept in contact with the mucosa of interest which allows for a resolution of 7–10 micrometers and an imaging depth of 2-3 mm depending on the wavelength of light used and the type of tissue being imaged [6, 7]. This allows visualization of histologic morphology in real time, especially the epithelial structures such as villi, crypts, and squamous and intestinal epithelium. In depth technical details and principles of optics involved in OCT have been discussed extensively elsewhere [8, 9].

Some of the more recent commercially available or custom-made OCT probes are Nvision VLE Imaging System (Nine Point Medical, Cambridge, MA) and probes from Lightlab Imaging (Westford, MA).

## 3. Gastrointestinal Applications

In the gastrointestinal tract, OCT has been used for imaging of the esophagus, stomach, small and large intestine, and biliary and pancreatic ducts. However, much of the experience and practical utility with OCT has been with esophageal, biliary, and pancreatic duct imaging.

Salient *in vivo* studies in the human gastrointestinal tract using OCT are summarized in Table 1.

## 4. Esophageal Imaging with OCT

OCT has been shown to demonstrate the five-layered esophageal wall with good correlation with histologic structures [10]. With newer advances in techniques for endoscopic mucosal resection (EMR) [11] and ablation (radiofrequency and cryotherapy), assessing the depth of invasion of mucosal cancers is vital, with a pivotal role for OCT. Indeed, studies have shown superiority of resolution for OCT compared to EUS specifically for visualization of the mucosa and submucosa [12].

OCT is of particular importance in imaging patients with Barrett's esophagus (BE). Patients with BE are at an increased risk for development of esophageal adenocarcinoma [13] and the incidence of esophageal adenocarcinoma has increased by 300–500% in white men in the last 30 years [14, 15].

**Figure 1:**
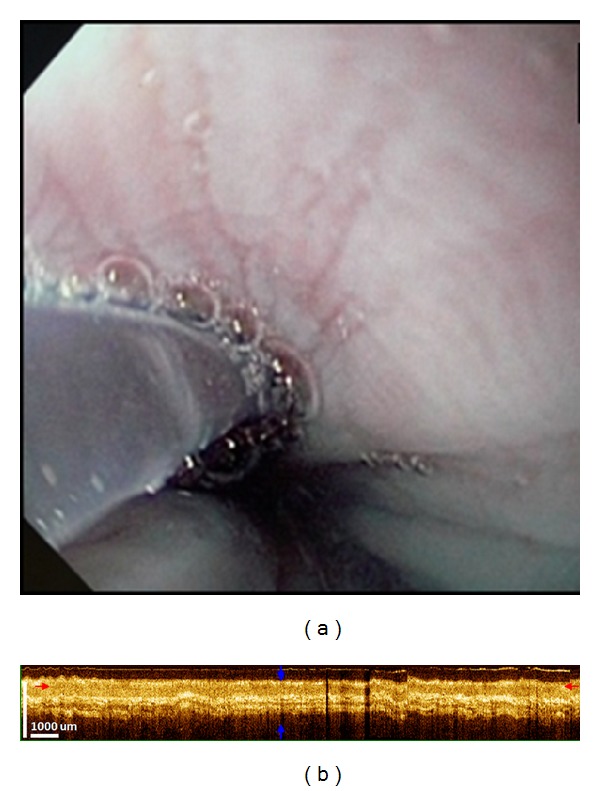
OCT imaging of normal esophagus. (a) Conventional endoscopy of the esophagus showing smooth pale mucosa. (b) Corresponding OCT image showing a well-defined, layered architecture. The epithelium, lamina propria, muscularis mucosa, submucosa, and muscularis propria are seen as distinct layers with alternating hypo- and hyperintensity.

**Figure 2:**
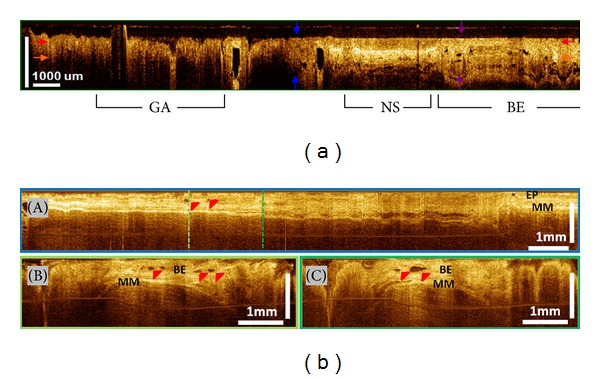
Barrett's esophagus (BE) without dysplasia. (a) Cross-sectional OCT imaging showing clear differences in layered architecture between gastric (GA), normal squamous (NS), and BE regions. BE regions exhibit distortion of the layered architecture and abnormal glandular features. (b) Cross-sectional OCT images around GEJ. BE glands (red arrows) are clearly observed (EP: epithelium; MM: muscularis mucosae in photos A–C).

**Figure 3:**
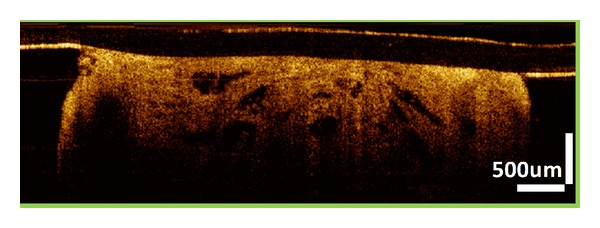
Intramucosal esophageal adenocarcinoma. OCT image showing dense large glands within the specimen. Lamina propria and muscularis mucosae (MM) layers are not clearly visible due to the infiltration of metaplasia into the MM layer.

The feasibility of OCT for carrying out *in vivo* real time imaging of Barrett's esophagus, high grade dysplasia and esophageal adenocarcinoma has been well demonstrated (Figures 1, 2, and 3). In their study using ultra-high resolution OCT, Chen and colleagues [16] demonstrated characteristic layered epithelium in a normal esophagus with normal architecture, while images of Barrett's esophagus corresponded to crypt-like glandular structures. High grade dysplasia and esophageal adenocarcinoma images exhibited more heterogeneous structures corresponding to irregular, heterogeneous tissue morphology from distorted and cribriform or villiform glandular architecture. A prospective study showed that OCT had a sensitivity of 68% and specificity of 82% with a diagnostic accuracy of 78% for detection of dysplasia in Barrett's esophagus [17]. In this study, Isenberg et al. used 314 pairs of OCT images and biopsy specimens from 33 patients and blinded four endoscopists and one pathologist to the histology results/real time OCT images and arrived at their findings using histology as a gold standard.

The current Seattle Protocol for surveillance for Barrett's esophagus recommends random 4 quadrant biopsies and leaves room for sampling error due to missed areas of dysplasia at random biopsies. OCT can be useful in guiding biopsies or eventually acquiring optical biopsies *in lieu* of standard biopsies. Each 3D-OCT data set provides approximately 160 mm^2^ (8 mm circumference × 20 mm pullback) coverage of the esophagus if the tissue is fully wrapped around the probe. This is approximately 30 to 60 times larger than the area sampled by jumbo biopsy forceps (~6 mm^2^) and standard biopsy forceps (~2.5 mm^2^) [18], thus, reducing sampling error.

OCT has also been used to show the surface morphology of rarer entities such as heterotopic gastric mucosa in the upper esophagus, also known as cervical inlet patch [19, 20].

**Figure 4:**
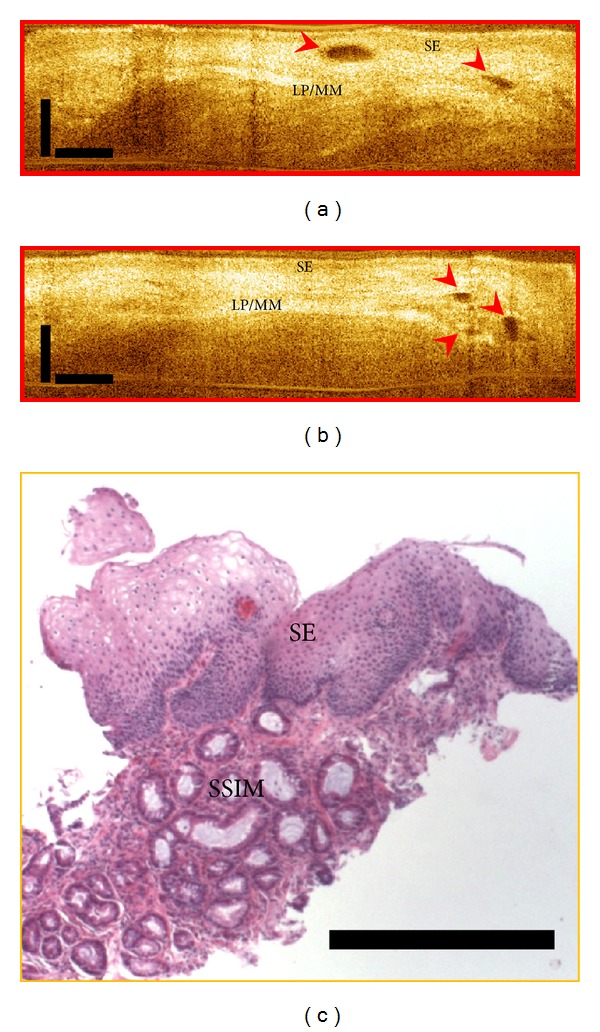
Subsquamous Intestinal Metaplasia (SSIM). (a-b): OCT images showing “buried glands” (red arrows). SE: squamous epithelium; LP/MM: lamina propria/muscularis mucosae. (c) Corresponding pathology showing subsquamous intestinal metaplasia under squamous epithelium.

An emerging utility of OCT can be in detecting Subsquamous Intestinal Metaplasia at the Gastroesophageal junction. Subsquamous Intestinal Metaplasia (SSIM) which has also been variably described as buried Barrett's glands or buried glands or subsquamous glands are areas of metaplastic columnar tissue present below normal appearing squamous mucosa (Figure 4). SSIM has been known to be present *de novo* [21, 22] in patients with BE and can persist in patients with BE after acid suppressive therapy and ablative therapies for Barrett's esophagus such as radiofrequency ablation, cryotherapy, or photodynamic therapy. Although the true malignant potential of residual SSIM is not known, concerns regarding identification and surveillance of SSIM are genuine owing to reports of progression to dysplasia and adenocarcinoma [23, 24]. Also, SSIM evades detection on conventional white light and narrow band endoscopy and can be missed even on biopsy using standard forceps due to sampling error and insufficient depth as shown by Gupta et al. [25]. Recently, one group working with OCT has demonstrated the existence of subsquamous intestinal metaplasia after radiofrequency ablation of Barrett's esophagus using OCT technology [18, 26]. This study showed regular flat squamous mucosa with small subepithelial vessels and glands in the normal esophagus. In contrast, BE showed large, densely packed glands with distortion of mucosal architecture. In post-RFA BE, findings were of a small number of isolated glands buried beneath 300–500 microns of neosquamous epithelium and lamina propria.

Going further, using OCT, it has been shown that the thickness of BE mucosa immediately after radiofrequency ablation predicts the response to RFA [27]. This study showed that BE mucosa was significantly thinner in patients who achieved complete eradication of intestinal metaplasia compared to those who did not achieve complete eradication of intestinal metaplasia at follow-up (257 ± 60 *μ*m versus 403 ± 86 *μ*m; *P* < 0.0001). A threshold thickness of 333 *μ*m derived from receiver operating characteristic curves corresponded to a 92.3% sensitivity, 85% specificity, and 87.9% accuracy in predicting the presence of BE at follow-up. These findings may have important implications for the need for more RFA sessions.

OCT has also been used to delineate the difference in architectural changes after different endoluminal ablative therapies for Barrett's esophagus. Radiofrequency ablation was observed to induce 230~260 micrometer depth of architectural changes after each set of ablations over a particular region, while cryotherapy was observed to induce edema-like spongiform changes to ~640 *μ*m depth [28].

## 5. OCT in the Small Intestine

There is limited data on the use of OCT in the small bowel. OCT has been used to image small intestinal mucosa and demonstrated 100% agreement with histology in a blinded study for differentiating between no atrophy and mild and marked atrophy of villous architecture [29]. This finding can be important to differentiate celiac disease from iron deficiency anemia in which villous architecture is typically preserved. An endoscopic doppler OCT has been used to show increased microvascularity in villi in duodenal adenomas [30].

## 6. OCT in the Colon

A number of studies have used OCT for evaluation of the large bowel. A study by Pfau et al. [31] showed that adenomas had significantly less structure and scattered light to a lesser degree than hyperplastic polyps and that hyperplastic polyps were significantly closer in organization and light scattering to normal mucosa as compared with adenomas. Other studies have characterized OCT findings in the normal colon, ulcerative colitis (UC), Crohn's disease (CD), and radiation proctitis [32–36]. The ability of OCT to image all the layers of the gastrointestinal wall can find utility in diagnosing the transmural inflammation of Crohn's disease (CD) and enable differentiating this from ulcerative colitis (UC). A prospective, blinded study by Shen and colleagues [37] showed a sensitivity of 90.0% and specificity of 83.3% for OCT in detecting the disrupted layered structure of the colon wall indicative of transmural inflammation, providing a valuable tool to distinguish CD from UC. This is especially relevant since biopsies are insufficient to assess for transmural inflammation.

## 7. OCT in the Biliary and Pancreatic Ducts

With the miniaturization of OCT probes, it is possible to use this technology for imaging the biliary and pancreatic ducts and evaluate strictures for neoplasia during ERCP. This was first demonstrated *in vivo* in the bile ducts by Seitz and colleagues in 2001 [38]. Their study demonstrated the layered architecture of the bile ducts similar to that found on histologic sections as well as underlying retroperitoneal structures with less backscattering. Similarly, OCT can recognize a differentiated three-layered architecture of the pancreatic duct in all cases with normal main pancreatic duct or chronic pancreatitis, whereas the layered architecture appears subverted in neoplastic lesions, with heterogeneous backscattering of the signal [39].

Given the low sensitivity (65%) of brush cytology for detection of malignancy in biliary strictures even in combination with other sampling techniques such as biopsy forceps [40], OCT offers a promising alternative. OCT has indeed been shown to enhance the yield of brush cytology for detection of malignant biliary strictures. Arvanitakis and colleagues [41] showed that the diagnostic sensitivity for biliary strictures could be increased to 84% by combining biliary brushings with 2 OCT criteria which were a disorganized and subverted layer structure and large hypo- or nonreflective areas considered as tumor vessels.

Testoni and colleagues performed a prospective study in 12 patients using OCT imaging with ERCP [42]. Twelve consecutive patients with documented main pancreatic duct stricture were investigated by endoscopic ultrasonography (EUS) and ERCP, followed by brush cytology and OCT scanning. OCT recognized a differentiated three-layer architecture in all cases with normal main pancreatic duct or chronic pancreatitis, while in all the neoplastic lesions the layer architecture appeared totally subverted, with heterogeneous backscattering of the signal. The accuracy of OCT for detection of neoplastic tissue was 100% compared with 66.7% for brush cytology.

## 8. Current Hurdles and Future Directions

At present, different OCT probes differ in their scanning speed, resolution, and depth penetration. There is a an unmet need for establishment of uniform objective and subjective criteria which can be used by the endoscopist for real time assessment of mucosal characteristics which can aid in differentiating normal from neoplastic tissue and identify varying grades of dysplasia. While OCT can easily identify intestinal metaplasia within a normal esophagus, its ability to identify dysplasia within Barrett's esophagus is relatively poor as shown in a prospective study by Isenberg and colleagues [17] and it calls for further improvements in imaging technique, such as involving computer aided image analysis which can identify textures and patterns indicative of dysplasia which may be underappreciated by the human eye. Efforts are underway in using computer aided image analysis for detection of dysplasia in Barrett's esophagus [43]. A consensus on the various terminologies used for imaging technologies would help standardize methods and findings and avoid ambiguity. Comparison of OCT with other imaging technologies is needed, and, most importantly, larger prospective data assessing clinical outcomes with OCT imaging is crucial which can identify niche areas where OCT can be sensitive, reliable, and have a high impact with respect to determining further therapy for patients. There are limitations to every new technology and identifying specific high yield applications for OCT will be required before it can be routinely used by practicing gastroenterologists.

## 9. Conclusions

OCT is a promising noninvasive imaging technology easily accessible through the working channel of an endoscope. OCT imaging has been performed in various parts of the GI tract, though mainly restricted to major academic and research institutions. Limitations of OCT include relatively high costs, need for standardized terminology and criteria for normal and neoplastic tissues, and lack of prospective data on clinical outcomes. With further refinement of this technology, OCT may allow “true optical biopsies” in the future.

## Figures and Tables

**Table 1:** *In vivo* OCT studies in the human gastrointestinal tract.

Year	Author	Number of patients	Anatomic location/pathology
1997	A. M. Sergeev	3	Esophagus, stomach
2000	B. E. Bouma	32	Barrett's esophagus
2000	S. Jäckle	22	Esophagus, stomach, colon
2000	M. V. Sivak Jr.	72	Esophagus, stomach, duodenum terminal ileum, colon, rectum
2000	X. D. Li	8	Esophagus
2001	U. Seitz	4	Bile ducts
2001	J. M. Poneros	121	Barrett's esophagus
2001	G. Zuccaro	69	Esophagus, stomach
2002	J. M. Poneros	5	Bile ducts
2004	B. Shen	70	Crohn's disease and ulcerative colitis
2005	Isenberg	33	Barrett's esophagus
2005	V. X. Yang	22	Esophagus, stomach, duodenum
2006	J. A. Evans	55	High grade dysplasia/intramucosal carcinoma in Barrett's esophagus
2006	P. A. Testoni	15	Pancreatic duct
2006	E. Masci	40	Celiac disease
2007	Y. Chen	50	Barrett's esophagus
2008	P. Consolo	35	Ulcerative colitis and Crohn's disease
2009	M. Arvanitakis	37	Biliary strictures
2009	D. C. Adler	4	Colon, ulcerative colitis, radiation proctitis
2010	W. Hatta	62	Superficial squamous cell esophageal cancer
2012	T. H. Tsai	13	Barrett's esophagus
2012	C. Zhou	1	Cervical inlet patch
